# Association of High Density Cholesterol With Hyperemic Epicardial Flow and Frame Count Reserve in Patients With Moderate Coronary Lesions and Slow Coronary Flow

**DOI:** 10.7759/cureus.13985

**Published:** 2021-03-18

**Authors:** Niya E Semerdzhieva, Stefan Denchev

**Affiliations:** 1 Emergency Department, National Heart Hospital, Sofia, BGR; 2 Department of Cardiology, Medical Center 'Mediva', Sofia, BGR

**Keywords:** frame count reserve, slow coronary flow, high density lipoproteins, verapamil

## Abstract

Background

Patients with microvascular angina and non-obstructive coronary atherosclerotic disease have an elevated risk of adverse events and all-cause mortality compared with individuals without ischaemic heart disease. The diagnosis coronary microvascular dysfunction in this setting relies on the detection of impaired coronary flow at rest or on calculation of coronary flow reserve. Previous studies demonstrate that the coronary flow reserve assessed by the corrected thrombolysis in myocardial infarction method - the frame count reserve is an objective quantitative alternative to other widely used invasive methods for microvascular status evaluation.

Purpose

We assessed the significance of clinical, hemodynamic, angiographic variables and therapy with reference to FCR in a small group of patients with up to moderate atherosclerotic coronary lesions and slow coronary flow. Materials and methods: Frame count reserve was evaluated in 15 patients without flow-limiting (>50%) coronary stenoses admitted with unstable angina. Frame count reserve was calculated by dividing the baseline corrected thrombolysis in myocardial infarction frame count (cTFC) by the cTFC assessed after intracoronary infusion of 100 µg of the calcium channel blocker - verapamil.

Results

The values of frame count reserve correlate positively with the levels of high density cholesterol (r= 0.900, p=0.001), inversely coronary flow after the application of verapamil - cTFCv (r= - 0.534, p=0.049). cTFCv was positively related with the levels of high density lipoproteins (r = - 0.645; p= 0.044) and was negatively influenced by the presence of atherosclerotic lesions at quantitative angiography (42.8±19.1 (n=8) vs 23±5.4 (n=7), p=0.029).The therapy with β-blocker and long-acting nitrate was associated with insignificantly higher frame count reserves after intracoronary verapamil compared to the continuous intake only of β-blocker or β-blocker and verapamil (2.1±0.78 vs 1.34±0.14 vs 1.70±0.70, p=NS).

Conclusions

Higher high-density lipoproteins relate to higher frame count reserves evaluated using verapamil. The improved blood flow after this microvascular vasodilator is consistently positively related to high-density cholesterol and the lack of atherosclerosis at conventional coronary angiography. The combined intake of micro- and macrovascular vasodilator could be associated with higher frame count reserves compared to therapy with β-blocker and one vasodilating drug.

## Introduction

In the absence of flow-limiting coronary atherosclerosis and macrovascular coronary spasm, the maximal flow to the heart in response to physical, emotional stress or pharmacological provocation is entirely dependent on the coronary microvascular resistance. Coronary flow reserve (CFR) could be reduced in patients with atherosclerotic risk factors, recurrent episodes of chest pain and mild or no coronary atherosclerotic lesions on conventional angiography [1-3]. The diagnosis coronary microvascular dysfunction in the patients without obstructive coronary disease relies on detection of angina, objective signs of myocardial ischemia and impaired coronary flow at rest or on calculation of CFR - the ratio of baseline coronary flow velocity to the velocity following intracoronary arteriolar vasodilator [4, 5]. The index of coronary microvascular function, obtained by the assessment of coronary flow velocity, using the corrected thrombolysis in myocardial infarction frame count (cTFC) method is known as frame count reserve (FCR) [6]. It has been demonstrated in previous studies that FCR is an objective quantitative alternative to other invasive techniques for microvascular status evaluation [6-8].

We assessed the significance of clinical, hemodynamic, angiographic variables and therapy with reference to FCR in a small group of patients with up to moderate atherosclerotic coronary lesions and slow coronary flow. 

## Materials and methods

FCR was evaluated during coronary angiography in 15 patients without flow-limiting (>50%) coronary stenoses admitted with unstable angina and non-ST elevation acute myocardial infarction to the Clinic of Cardiology of University Hospital ‘Alexandrovska’ in the period 2006-2008. The angiographic study was performed in the morning. The medical therapy was not discontinued before angiography. During the examination, a 6Fr diagnostic catheters and nonionic contrast medium (iopamidol 370) were used. The coronary diameters were measured 5 mm distal to the tip of the diagnostic catheter. The normal epicardial caliber of the artery with delayed coronary flow was used for this analysis based on the averaged value of two or three diastolic measurements in an angiographic view in which the respective artery could be optimally visualized. Coronary flow velocity during coronary angiography was measured applying the corrected thrombolysis in myocardial infarction method frame count method, TFC [9]. TFC was corrected for the length of left anterior descending coronary artery (LAD) by dividing of TFC measured in LAD by 1.7 and this index-corrected TFC (cTFC) was used. FCR was calculated by dividing the baseline cTFC by the cTFC following intracoronary infusion of 100 μg of the calcium channel blocker verapamil. The hyperemic cTFC was evaluated with a repeat angiogram performed 2 minutes after verapamil. The cTFC were analyzed by one angiographer, and the angiograms were recorded at 12.5 frames/s. A coronary flow of cTFC>27 frames was defined as abnormally slow. The change in coronary artery diameter after the injection of verapamil expressed as a percent change from the baseline diameter were also reported.

In five patients (33.3%), the coronary angiography was performed as an emergency procedure. The rest of the study group had been referred for angiography following symptoms associated with dynamic changes in stress ECG, Holter ECG or in a resting ECG consistent with myocardial ischemia. Ten of the patients (66.6%) had been taking statin for 20.5±10.3 days (10÷30 days) before the angiography; 13 patients (86.7%) had used combined vasoactive drug therapy for at least 15.4±12.3 days (2÷30 days) prior to admission and continued with this therapy in the hospital. In two patients, β-blocker and nitrate were started at admission. In one patient the angiographic study was performed without therapy because of a tendency for medication-related bradycardia and hypotension and also because a symptom relief was achieved by the time of the angiographic procedure. The levels of plasma cholesterol were assessed at admission.

The systolic function of left ventricle was evaluated by calculating the ejection fraction at echocardiography by applying the Simpson's method. Left ventricular hypertrophy was defined as thickness of interventricular septum or left ventricular posterior wall in the end-diastole equal or exceeding 12 mm. The left ventricular mass was calculated using the Cube formula based on the linear measurements of internal left ventricular diameter - LVID, inferolateral posterior wall thickness (PWT) and thickness of interventricular septum (IVS):

Left ventricular mass = 0.8x1.04x[(IVS+LVID+PWD)3 - LVID3]+0.6 g

For each patient, the left ventricular mass was recalculated as an index of the body surface area and afterwards, included in the analysis.

Epicardial coronary artery stenoses >50%, ostial lesions of any severity in the main coronary arteries, previous coronary revascularization procedures, left ventricular systolic dysfunction (defined as ejection fraction <50%), wall motion abnormalities at rest, valve prolapse, intermediate and severe valve stenosis or insufficiency, clinical or laboratory signs of infectious disease or intake of antibiotics, and also suboptimal angiographic imaging were exclusion criteria for the study.

The subgroup of patients without atherosclerotic lesions causing less than 40% coronary stenosis, with slow flow (cTFC>27 frames) in at least one coronary artery in the absence of left ventricular hypertrophy, coronary aneurysms, ectasia, coronary fistulas, myocardial bridges formed the group characterized as slow coronary flow phenomenon (SCFP). Coronary artery ectasia was defined as multiple saccular, or fusiform distensions of parts of a coronary vessel of up to one and a half times the diameter of an adjacent normal segment. 

Data were analyzed using SPSS software, version 19 (SPSS Inc., Chicago, IL). Categorical variables wеre presented as counts and percentages. Chi-square test or Fisher’s exact test were used for comparisons of categorical variables. Continuous variables were presented as mean and standard deviation (SD). Normality of the continuous variables was analyzed with the Shapiro-Wilk test. Depending on the result of Levene’s test, variables with normal distribution were compared with the Student test or Welch test. If the normality test failed, variables were compared using the Mann-Whitney U-test. The paired t-test was applied in examining the difference between repeated measurements. Parametric and non-parametric correlation analysis was used for the assessment of the relation of two continuous variables. Analysis of variance (one-way ANOVA) was used for the analysis of the effects of therapy on FCR. P-values lower than 0.05 were considered statistically significant.

All patients have signed written informed consents for the performance of the invasive procedures. This retrospective analysis received an assessment by the committee of ethics of University Hospital ‘Alexandrovska’ and was approved as not being in conflict with the regulations for research involving humans and reporting medical information.

## Results

All patients had impaired coronary flow at rest. Four patients (26.7%) were diagnosed with SCFP, coronary aneurysms were found in two patients (13.3%). The rest of the study group (60%, n=9) were patients with coronary stenosis < 50% and hypertensive heart disease. Slow flow in one, two and all three coronary arteries was diagnosed respectively in 7 (43.8%), 3 (18.8%) and 5 (31.3%) patients. The average values FCR were 1.88±0.6, 0.96÷3.1 (Table 1). The average age of the study group 57.3±8.6 years represented a significant indicator of coronary disease. Most of the patients (60%) were women (Table 2). Arterial hypertension and dyslipidemia were prevalent risk factors for atherosclerosis in the study cohort. Almost half of the group were mildly obese, minority suffered diabetes mellitus. Data regarding smoking was available in 5 (33.3%) patients. Two were ex-smokers, the rest were non-smokers. Echocardiographic signs of left ventricular hypertrophy (LVH) were found in two-third of the patients. LVH was mild with for almost all these patients. The left ventricular mass was 112.7±17.5 (75÷145) g/m^2^. Chest pain and/or ischemic ECG deviation could be induced at stress electrocardiography in substantial number of patients (Table 3).

**Table 1:** Clinical, angiographic, echocardiographic and laboratory data. FCR: frame count reserve; cTFC and cTFCv: corrected thrombolysis in myocardial infarction frame count at baseline cTFC and after coronary verapamil; LV wall: maximal thickness of left ventricular posterior wall or interventricular septum; TChol: plasma levels of total cholesterol; HDL: plasma levels of high-density lipoproteins; LDL: plasma levels of low-density lipoproteins; TG: plasma levels of triglycerides; BMI: body mass index; BSA: body surface area.

Variable	Mean ± SD	p	Median	Minimum	Maximum
Age, years	57.3±8.6	0.175	59	45	75
FCR	1.88±0.60	0.313	1.52	1	3
cTFC, frames	55.9±27	0.022	44	30	118
cTFCv, frames	34.9±17.9	0.015	28	14	82
LV wall, mm	11.7±0.8	0.055	12	10	13
TChol, mmol/l	5.4±1.1	0.164	5.1	4	8
HDL, mmol/l	1.3±0.2	0.129	1.3	1	2
LDL, mmol/l	3.0±0.8	0.231	2.8	2	5
TG, mmol/l	1.8±0.7	0.302	1.9	1	3
BMI, kg/m^2^	30.6±3.4	0.905	30	25.3	36
BSA, m^2^	1.9±0.2	0.199	2	1.72	2.19

 

**Table 2:** Angiographic characteristics of the patients. Dves, Dvesv: epicardial coronary diameter at baseline and after verapamil; cTFC, cTFCv: corrected TIMI frame count before and after verapamil; LAD: left anterior descending branch of left coronary artery; RCx: circumflex branch of left coronary artery; RCA: right coronary artery; RIM: intermediate branch of left coronary artery; D1: first diagonal branch of left coronary artery.

Variable	Age, years	Gender	Artery	Dves, mm	Dvesv, mm	cTFC, frames	cTFCv, frames	Atherosclerosis
Patient 1	45	Female	LAD	4.4	4.4	38	25	
Patient 2	75	Male	LAD	3.3	3.6	37	27	Distal branch of RIM – 45%-50% stenosis
Patient 3	68	Female	RCx	2.9	3.3	82	66	
Patient 4	64	Female	RCx	3.6	4	101	82	LAD, RCx - irregularities, ectasia
Patient 5	57	Male	RCA	4	4	43	19	D1 - 40% stenosis
Patient 6	50	Male	LAD	3.3	3.7	35	28	
Patient 7	62	Female	LAD	3.6	4.6	30	31	LAD, RCA - diffuse thickening
Patient 8	54	Female	LAD	4	4.3	38	14	
Patient 9	48	Female	LAD	4.5	4.5	65	28	
Patient 10	59	Male	RCA	4.3	4.3	115	38	
Patient 11	61	Female	LAD	4	4	44	28	D1 - 40% stenosis
Patient 12	62	Female	LAD	4.8	4.4	47	25	
Patient 13	48	Male	LAD	4.3	4.3	120	48	
Patient 14	60	Female	LAD	3.1	3.3	34	25	Irregularities
Patient 15	47	Male	LAD	4.8	5.2	79	35	

**Table 3:** Demographic characteristics, cardiovascular risk profile. LVH: left ventricular hypertrophy; stress ECG: exercise stress electrocardiographic test; β-blocker + CCB: therapy with β-blocker and calcium channel blocker; β-blocker + N: β-blocker and long-acting nitrate.

Variable	N (%)
Women/men	6 (40)/9 (60)
Arterial hypertension	15 (100)
Dyslipidemia	13 (86.7)
Diabetes mellitus	3 (20)
Obesity	10 (66.7)
LVH	10 (66.7)
Positive stress ECG /n=9/	8 (88.9)
Atherosclerotic plaques	6 (40)
No anti-ischemic drug	1 (6.7)
β-blocker	6 (42.9)
β-blocker + CCB	4 (28.6)
β-blocker + N	4 (28.6)

Most of the patients were on lipid-lowering therapy with statin 93.3 (n=14). The intake of angiotensin-converting enzyme (ACE-I) and aspirin or clopidogrel in the patient group were 73.3% (n=11) and 66.7% (n=10), respectively. Coronary atherosclerosis at quantitative coronary angiography was detected in six patients - three patients had only irregularities, two patients presented with plaques causing 40% coronary narrowing and one with coronary stenosis of 50%.

Marked differences in the values of cTFC, coronary lumen diameter and systolic blood pressure were found following the intracoronary injection of 100 µg verapamil compared to these parameters at baseline. The heart rate of study patients did not change significantly after verapamil. These results are shown in Table 4.

**Table 4:** Angiographic and clinical variables before and after intracoronary verapamil application. cTFC: corrected thrombolysis in myocardial infarction frame count; Dves: epicardial vessel diameter; SBP: systolic blood pressure; HR: heart rate.

Variable	Before verapamil	After verapamil	Δ	p
Dves, mm	3.9±0.6	4.2±0.6	-0.3	0.009
cTFC, frames	53.9±27	34.9±17.9	21.7	0.001
SBP, mmHg	124.3±20.1	119.4±18.2	6.6	0.025
HR, bpm	68 ±7.6	65.5±11	2.5	0.493

The mean epicardial vasodilation after verapamil was 5.1±4.8% (0÷12.2%). Six patients (40%) showed no vasodilation (0%) in response to verapamil.

The values of the frame count reserve in the subset of patients with dyslipidemia, in those with diabetes, also with pathologic stress ECG and of the female patients were insignificantly lower but with pronounced difference compared FCR of the rest (Table 5).

**Table 5:** Association of FCR with cardiovascular risk indicators. LVH: left ventricular hypertrophy; stress ECG: exercise stress electrocardiographic test; ACE-I: therapy with angiotensin-converting enzyme inhibitor; FCR: frame count reserve.

	n	FCR		p
Variable		-	+	
Men/women	6/9	1.91±0.76	1.64±0.57	NS
Dyslipidemia	2/13	2.49±0.32	1.66±0.62	NS
Diabetes mellitus	12/3	1.94±0.64	1.21±0.3	NS
Smoking	3/2	1.94±0.74	2.68±0.59	NS
Obesity	4/10	1.61±0.46	1.85±0.73	NS
LVH	5/10	1.79±0.68	1.78±0.68	NS
Positive stress ECG	1/8	2.32	1.61±0.8	NS
Atherosclerotic plaques	9/8	1.67±0.72	1.86±0.54	0.584
ACE-I	4/11	1.68±0.49	1.77±0.71	NS

Greater FCR after intracoronary verapamil were measured in the patient group on therapy with β-blocker and nitrate (β-blocker vs β-blocker and calcium channel blocker vs β-blocker and nitrate - 1.70±0.70 vs 1.34±0.14 vs 2.1±0.78, p=NS). Angiographic or hemodynamic variables did not differ with regard to the type of the anti-ischemic drug combination used.

We observed a significant positive association with high-density lipoprotein cholesterol with FCR (r=0.900, p=0.001) and a negative correlation of the cTFC after verapamil with FCR (r=-0. 534, p=0.049). Substantially lower FCR (although statistical significance in this study group) could be found in the patients with dyslipidemia, diabetes and also in women. The association of FCR with clinical variables in the study population are presented in Table 6.

**Table 6:** FCR - correlation with demographic, angiographic, hemodynamic parameters and the lipid levels. cTFC: corrected TIMI frame count; cTFCv: corrected TIMI frame count after intracoronary injection of verapamil; Dves: epicardial coronary lumen diameter; Dvesv: epicardial coronary lumen diameter after intracoronary injection of verapamil; HR: heart rate; HRv: heart rate after intracoronary injection of verapamil; SBP: systolic blood pressure; SBPv: systolic blood pressure after intracoronary injection of verapamil; TChol: plasma levels of total cholesterol; HDL: plasma levels of high-density lipoproteins; LDL: plasma levels of low-density lipoproteins; TG: plasma levels of triglycerides; BSA: body surface area; LV wall: maximal left ventricular wall thickness; BSA: body surface area.

FCR	r	p
Age	-0.325	0.259
cTFC	0.260	0.370
cTFCv	-0.534	0.049
Dves	0.416	0.139
Dvesv	0.108	0.714
SBP	0.109	0.735
SBPv	0.043	0.895
HR	0.335	0.263
HRv	-0.091	0.766
TChol	-0.162	0.654
HDL	0.900	0.001
LDL	0.062	0.874
TG	-0.569	0.110
LV wall	0.054	0.855
Myocardial mass	0.189	0.500
BSA	0.014	0.963

Further analysis showed that HDL cholesterol was consistently related to improved blood flow after microvascular vasodilator and was as tendency reduced in the patients with diabetes mellitus (Table 7 and Table 8). cTFCv correlated positively with borderline significance with the baseline cTFC, was associated with the presence of atherosclerotic plaques at quantitative coronary angiography and it was substantially although not significantly greater in the patients with left ventricular hypertrophy, dyslipidemia and positive stress tests (Table 9 and Table 10).

**Table 7:** HDL association with risk profile, therapy with statin and risk of ischemia. Stress ECG: exercise stress electrocardiographic test; HDL: high-density lipoprotein.

	HDL mmol/l		p
Characteristic	-	+	
Men/women	1.3±0.2	1.3±0.2	NS
Dyslipidemia	2.5±0.3	1.6±0.6	NS
Diabetes mellitus	1.4±0.2	1.2±0.1	NS
Obesity	1.3±0.1	1.3±0.2	NS
Positive stress ECG	1.2±0.02	1.6±0.4	NS
Atherosclerotic plaques	1.3±0.1	1.5±0.1	NS
Statin	1.5	1.3±0.2	NS

**Table 8:** Correlates of plasma HDL cholesterol. HDL: plasma levels of high-density lipoproteins; cTFC and cTFCv: corrected thrombolysis in myocardial infarction frame count at baseline and after intracoronary injection of verapamil; Dves and Dvesv: epicardial coronary lumen diameter before and after intracoronary injection of verapamil; TChol: plasma levels of total cholesterol; LDL: plasma levels of low-density lipoproteins; TG: plasma levels of triglycerides; BMI: body mass index.

HDL	r	p
Age	0.030	0.933
cTFC	-0.148	0.688
cTFCv	-0.645	0.044
Dves	0.530	0.115
Dvesv	0.133	0.714
TChol	-0.317	0.327
LDL	-0.244	0.497
TG	-0.451	0.191
BMI	-0.011	0.976

**Table 9:** Baseline cTFC after verapamil correlation with angiographic and hemodynamic variables. cTFC: corrected TIMI frame count; cTFCv: corrected TIMI frame count after intracoronary injection of verapamil; Dves: epicardial coronary lumen diameter; Dvesv: epicardial coronary lumen diameter after intracoronary injection of verapamil; SBP: systolic blood pressure; SBPv: systolic blood pressure after intracoronary injection of verapamil; HR: heart rate; HRv:  heart rate after intracoronary injection of verapamil; TChol: plasma levels of total cholesterol; HDL: plasma levels of high-density lipoproteins; LDL: plasma levels of low-density lipoproteins; TG: plasma levels of triglycerides.

	n	cTFCv, frames		p
Characteristic		+	+	
Men/women	6/9	31.5±8.3	37.1±22.4	NS
Dyslipidemia	2/13	24.5±14.9	36.5±18.3	NS
Diabetes mellitus	12/3	34.7±19.8	35.7±9.2	NS
Obesity	5/8	36.4±17	34.1±19.1	NS
Positive stress ECG	1/8	28	36.5±13.9	NS
Atherosclerotic plaques	8/7	42.8±19.1	23±5.4	0.029
LVH	5/10	27±9.6	38.8±20.1	NS
ACE-I	4/11	32±7.4	35.9±20.6	NS

**Table 10:** Baseline cTFC after verapamil correlation with angiographic and hemodynamic variables. cTFCv: corrected TIMI frame count after intracoronary verapamil; cTFC: corrected TIMI frame count; Dves: epicardial coronary lumen diameter; Dvesv: epicardial coronary lumen diameter after intracoronary verapamil; SBP: systolic blood pressure; SBPv: systolic blood pressure after verapamil; HR: heart rate; HRv: heart rate after intracoronary injection of verapamil; TChol: plasma levels of total cholesterol; HDL: levels of high-density lipoproteins in plasma; LDL: low-density lipoproteins; TG: plasma triglycerides.

cTFCv	r	p
Age	0.254	0.360
cTFC	0.505	0.055
Dves	-0.262	0.346
Dvesv	0.129	0.647
SBP	-0.113	0.726
SBPv	-0.071	0.827
HR	-0.213	0.485
HRv	0.066	0.932
TChol	0.432	0.396
LDL	0.302	0.396
TG	0.127	0.728

## Discussion

Studies of other investigators show that FCR provide a good estimate of coronary flow reserve measured using well-established methods [6, 7]. Previous research works that focus on assessment of CFR with the FCR method process data of either patients with obstructive coronary disease undergoing percutaneous coronary intervention [8] or of non-homogenous cohorts of subjects with non-obstructive or intermediate lesions mixed with patients with significant obstructive coronary disease [6,7]. To our knowledge, our analysis is the first to report results for FCR of population with non-obstructive atherosclerosis and severe coronary microvascular dysfunction characterized by slow coronary flow at rest. In this setting, almost a quarter of the patients were markedly reduced FCR values. The FCR in these cases was very close to the FCR of subjects with symptoms and signs of myocardial ischemia obstructive coronary disease prior to revascularization [6]. Considering the presence of coronary microvascular dysfunction, not surprisingly the FCR in more than half of the study group remained slightly lower than the immediate post-percutaneous intervention FCR in the setting of single and two-vessel coronary disease [7]. The FCR of >60% of our patients was clearly diminished compared to patients with intermediate coronary luminal narrowings without the potential to cause ischemia [7].

A main finding derived by the analysis of this small patient cohort is that the values of FCR correlate positively with HDL. We found a pronounced although insignificant difference - lower FCRs in the subgroups of patients with dyslipidemia, diabetes mellitus and in women in contrast to the male patients and the patients without the abovewritten pathologies. Substantially lower levels of HDL were observed in the diabetics. The result is consistent with previous reports on coronary flow reserve in absence of obstructive coronary disease [3]. In early stages of coronary atherosclerosis, dyslipidemia as a single atherosclerotic risk factor may cause myocardial capillary rarefication, increased microvascular resistance at rest and reduced coronary flow reserve [10,11]. We have found no data in the literature on direct comparison of the significance of separate atherosclerotic risk factors for vascular dysfunction development. Nevertheless, dyslipidemia is a powerful contributor to the process of atherosclerosis. By interacting with certain genetic variants (e.g., responsible for biosynthesis of nitric oxide) hypercholesterolemia significantly increases the risk of accumulation of atherosclerotic co-morbidities, such as hypertension, independently from the impact of age, sex, smoking status and body mass index [12]. Severe dyslipidemia may cause impairment in coronary blood flow as a result of reduction in the aortic elastic properties before the occurrence of clinical manifestations of atherosclerotic disease [13]. Higher reactive oxygen species and circulating oxidized LDL induce endothelial cell apoptosis and disregulation of vascular function [14-16]. Hypercholesterolemia itself can directly cause attenuation of the endothelium-dependent vasodilatation in coronary arterioles through impairing the coupling of adenylyl cyclase with potassium channels [17]. These are only a few of the possible mechanisms for microvascular abnormalities associated with dyslipidemia. On the contrary high HDL cholesterol is well-recognized protective factor regarding the process of atherosclerosis. HDL in tissues act as a reservoir for lipid peroxides generated on LDL, chelate metal ions and inhibit their ability to catalyse the peroxidation of lipids. HDL also carries several proteins with antioxidant activity (apolipoprotein A1, lecithin: cholesterol acyltransferase, paraoxonase) [18].

In the presented patient population, a consistent positive correlation of FCR with the cTFC after intracoronary verapamil - cTCFv was observed. Accordingly, in a cohort with insignificant coronary atherosclerosis and calculated mean FCR 2, Kim et al have demonstrated that the presence of greater hyperemic TFC induced by nitroprusside were significantly higher in the patients with microvascular angina than in controls [19]. The lower cTFC after the injection of verapamil reflect relatively preserved epicardial coronary vasodilation and greater change in the coronary flow velocities and greater FCR, respectively. An impaired response to vasodilators of both conduit arteries and arterioles would result in smaller coronary arterial area for a given amount of myocardium to be perfused [20]. Although our patients on average presented with percentage of epicardial vasodilation in response to intracoronary verapamil similar to that of healthy controls [21], in more than one-third the coronary macrovascular dilation was impaired - epicardial lumen did not change after verapamil. Unrecognized diffuse intimal coronary thickening and also coronary arterial remodeling may provide mechanisms for impaired vasodilation and reduction of coronary flow in the presence only of visual stenosis of < 50% on quantitative coronary angiography [22].

The discordance between anatomical and physiological severity of coronary atherosclerosis is also influenced by downstream myocardial mass to anatomical coronary stenosis. In our study, cTFCv were greater as tendency in the patients with dyslipidemia, with abnormal stress ECG tests and with left ventricular hypertrophy. In recent studies accordingly, a lower mean epicardial coronary arterial lumen volume and higher left ventricular myocardial mass and their ratio, have been associated with the presence of microvascular dysfunction and myocardial ischemia in population with insignificant coronary disease [23]. Despite some existing controversies, minimal coronary arteriolar resistance increases equally to the volume of myocardial fibrosis and to medial area identically in all patients with angina and atherosclerotic risk factors [24,25]. We have neither histologic quantification of myocardial fibrosis nor consistent available data regarding left ventricular diastolic function in this particular patient subgroup. The majority of our patients have left ventricular hypertrophy, probably entailing significant myocardial fibrotic depots and arterial remodeling.

In the present study, FCR was related to the coronary flow velocities after vasodilating agent but demonstrated a lack of association with the other coronary flow parameters, the systolic blood pressure and the heart rate before and after its intracoronary injection. The percentage change in thrombolysis in myocardial infarction frame count following coronary vasodilator or the FCR have been unrelated to systolic blood pressure and heart rate in earlier studies [8, 26], despite reported significant correlations of cTFC at rest with hemodynamic variables [27,28]. In our study, in contrast to some prior analyses, the post-drug change in heart rate is very small. Moreover, because of the utilization of bradycardic vasodilator such as verapamil and not suspending the anti-tachycardic therapy, the baseline and hyperemic heart rate in our patients remained low. This could be a possible explanation for insignificant relationship of FCR and the heart rate.

In the small patient cohort being analyzed, the usage of two vasodilators (intracoronary verapamil applied at the background therapy with oral nitrate) specifically inducing dilation in coronary arteries of different types as compared to a single vasodilator intake (calcium channel blocker) tend to be related to higher FCR. Similarly, previous studies underline the importance of a treatment regime, combining at least two different classes of vasodilators for controlling symptoms in patients with microvascular angina especially for cases with severely reduced coronary flow reserve [29,30].

Study limitations

The small sample size is a major limitation of our investigation, and thus our results should be considered uncertain. The lack of comparison with the invasive Doppler measurement of coronary flow velocity and reserve could also represent a disadvantage of the performed analysis. The etiology of coronary microvascular dysfunction was not thoroughly characterized with the use of specific methods (cardiac magnetic resonance imaging, provocation of coronary spasm during the invasive studies, etc.). The study was conducted without temporary stopping therapy, thus making the coronary flow velocity assessed with the TFC method relatively dependent on the effect of therapy on hemodynamic and angiographic (coronary lumen diameter) parameters. The epicardial vasodilation in response to the intracoronary verapamil might also be reduced because of the chronic medication.

## Conclusions

In summary, our analysis demonstrates that higher high-density lipoproteins relate to higher frame count reserve evaluated using verapamil. The improved blood flow after this microvascular vasodilator is consistently positively related to high-density cholesterol and the lack of atherosclerosis at conventional coronary angiography. The combined intake of micro- and macrovascular vasodilators could be associated with higher frame count reserves compared to the therapy with β-blocker and one vasodilating drug.
